# Nutritional Status and Recurrent Major Cardiovascular Events Following Acute Myocardial Infarction—A Follow-Up Study in a Primary Percutaneous Coronary Intervention Center

**DOI:** 10.3390/nu16071088

**Published:** 2024-04-08

**Authors:** Maria Czinege, Vasile-Bogdan Halațiu, Victoria Nyulas, Liliana-Oana Cojocariu, Bianca Ion, Violeta Mașca, Constantin Țolescu, Theodora Benedek

**Affiliations:** 1 Doctoral School of Medicine and Pharmacy, “George Emil Palade” University of Medicine, Pharmacy, Science and Technology of Târgu Mureș, 540139 Târgu Mureș, Romania; maria.czinege@umfst.ro (M.C.); biancapopovici12@gmail.com (B.I.); violetamasca@gmail.com (V.M.); 2 Department of Physiology, “George Emil Palade” University of Medicine, Pharmacy, Science and Technology of Târgu Mureș, 540139 Târgu Mureș, Romania; 3 Clinic of Cardiology, County Emergency Clinical Hospital, 540136 Târgu Mureș, Romania; cjrliliana@yahoo.com (L.-O.C.); cristi.tolescu95@gmail.com (C.Ț.); 4 Department of Informatics and Medical Biostatistics, “George Emil Palade” University of Medicine, Pharmacy, Science and Technology of Târgu Mureș, 540139 Târgu Mureș, Romania; victoria.rus@umfst.ro; 5 Department of Cardiology, “George Emil Palade” University of Medicine, Pharmacy, Science and Technology of Târgu Mureș, 540139 Târgu Mureș, Romania; theodora.benedek@umfst.ro

**Keywords:** major cardiovascular adverse events, nutritional status, acute myocardial infarction

## Abstract

Background: Acute myocardial infarction is often accompanied by malnutrition, which is associated with an imbalance between catabolic and anabolic processes. This ultimately leads to cardiac cachexia, which worsens the patient’s prognosis. We aimed to assess the correlation between nutritional status, assessed using the controlling nutritional status (CONUT) score, and the rate of major cardiovascular adverse events (MACE). Methods: The present investigation was a non-randomized, prospective, observational study in which 108 patients with acute myocardial infarction were included. Nutritional status was assessed using the CONUT score. Based on the CONUT score, the patients were divided as follows: Group 1—normal or mild nutritional status (CONUT < 3 points, *n* = 76), and Group 2—moderate to severe nutritional deficiency (CONUT ≥ 3 points, *n* = 32). Demographic, echocardiographic, and laboratory parameters were obtained for all patients, as well as the MACE rate at 1 and 3 months of follow-up. Results: The MACE occurred more frequently in patients with impaired nutritional status at both 1-month follow-up (46.9% versus 9.2%; *p* < 0.0001) and 3-month follow-up (68.8% versus 10.5%; *p* < 0.0001). In terms of cardiovascular events, patients with poor nutritional status, with a CONUT score ≥ 3, presented more frequent non-fatal myocardial infarction, stroke, revascularization procedure, and ventricular arrhythmia. Also, the number of cardiovascular deaths was higher in the undernourished group. Conclusions: This study found that patients with poor nutritional status experienced inflammatory status, frailty, and cardiovascular events more often than those with normal nutritional status at 1-month and 3-month follow-up after an acute myocardial infarction.

## 1. Introduction

Cardiovascular disease continues to be the leading cause of death in the European Union, accounting for up to 33% of deaths [1]. Modifiable risk factors, such as an unhealthy lifestyle, may play a role in preventing up to 80% of cardiovascular deaths [2]. Malnutrition is not unusual in cardiovascular patients, being associated with altered clinical status and a worse prognosis [3]. In a study published by Basta et al. [4], nearly 55% of patients with ST-segment elevation myocardial infarction (STEMI) were malnourished and presented lower long-term survival than those with normal nutritional status. In patients suffering an acute myocardial infarction (AMI), identification of a malnourished status is extremely important, since early correction of malnutrition may reduce the risk of complications, rehospitalization, and death [5].

Several tools have been developed to screen the nutritional status of patients on admission to the hospital, including the Nutrition Status Control Index (CONUT), the Geriatric Nutrition Risk Index (GNRI), and the Prognostic Nutrition Index (PNI), all of which are highly effective for early detection of malnutrition [6]. The CONUT score is calculated by serum albumin concentration, total peripheral lymphocyte count, and total cholesterol concentration, being the most commonly used screening tool for early detection of poor nutritional status [7]. In various studies, CONUT score was shown to have a strong association with patient prognosis and the presence of a cardiovascular disease, including coronary heart disease and congestive heart failure [8,9]. Although malnutrition has been reported to be associated with a high mortality rate in coronary heart disease and heart failure, little is known about the relationship between malnutrition and adverse cardiovascular events in patients with AMI in the post-infarction period [10]. Myocardial infarction poses an increasing burden on healthcare systems as its incidence is steadily rising and is responsible for a large proportion of deaths and reduced quality of life [11]. Both in-hospital and 1- and 3-month follow-up outcomes of patients admitted with AMI remain unsatisfactory due to particularly high mortality rates, reaching up to 8% at 3-month follow up according to Choles A.H. et al. [12].

Chronic cardiovascular diseases can lead to metabolic changes in the body, including alterations in glucose metabolism and increased energy expenditure [13]. These changes can affect the body’s nutritional requirements. Due to this, patients with AMI can be accompanied by an altered nutritional status and frailty, which is associated with an imbalance between catabolic and anabolic processes. This ultimately leads to cardiac cachexia, which worsens the patient’s prognosis [14,15]. On the other hand, both AMI and nutritional deficiency are associated with an increased inflammatory status; persistence of high inflammation in the postinfarction period may have a detrimental effect not only on the ventricular function, but also in the patient’s prognosis [16,17,18].

In patients with an acute coronary event, the prevention of a major acute cardiovascular event (MACE) represents an extremely important objective. The MACE serves as a comprehensive measure to evaluate the efficacy of treatments or interventions in reducing major cardiovascular events such as cardiovascular death, non-fatal MI, non-fatal stroke, unstable angina, or revascularization procedures [19]. The link between nutritional status and the rate of MARE in post-AMI patients is not yet fully understood.

The aim of this study was to investigate the correlation between nutritional status, assessed using the CONUT score, and the rate of MACE in patients following an AMI. We investigated as well the association between nutritional status and inflammatory status, assessed by highly sensitive *C*-reactive protein (hsCRP) and left ventricular function assessed by *N*-terminal B-type natriuretic peptide (NTproBNP), and left ventricular ejection fraction (LVEF) at echocardiography.

## 2. Materials and Methods

This was a non-randomized, prospective, observational study, including a total of 156 consecutive patients with AMI who received primary percutaneous coronary intervention in the Târgu Mureș Emergency County Clinical Hospital between 1 March 2023, and 15 May 2023. A total of 41 patients with pre-existing congestive heart failure, malignancy, chronic renal failure, nephrotic syndrome, liver failure, hematological diseases, autoimmune diseases, and rheumatological diseases were excluded to avoid bias related to the impact of these comorbidities on nutritional status. A total of 7 patients without available pre-procedural albumin, required for assessment of nutritional score, were also excluded from the study. The present study was approved by the Research and Ethics Committee of the Târgu Mureș Emergency Clinical County Hospital (Ad. 2159/10 February 2023) and the “George Emil Palade” University of Medicine, Pharmacy, Sciences and Technology of Târgu Mureș (2112/24 February 2023) and was conducted in accordance with the ethical principles set out in the Helsinki Declaration of 1975. All patients signed written informed consent after being informed about the study protocol, and all data were anonymized during analysis.

All clinical data, including age, gender, height, weight, body mass index (BMI), history of coronary heart disease, hypertension, stroke, and diabetes mellitus were collected from patient profiles. Biochemical data collected included albumin, urea, creatinine, uric acid, aspartate aminotransferase (GOT), alanine aminotransferase (GPT), cholesterol and its fractions, triglycerides, creatine kinase, ionogram, gamma-glutamyltransferase (GGT), leukocytes, etc. All these parameters were repeated at 1 month and 3 months after discharge from the hospital. Inflammatory status was assessed on the basis of serum hsCRP, determined at admission using the PATHFAST™ (Polymedco, Cortlandt, NY, USA) system, and repeated after 5 days. NTproBNP was determined to assess ventricular function and was repeated at 1 month and 3 months follow-up, using the same PATHFAST™ equipment.

The CONUT score was calculated using serum albumin levels, lymphocyte counts, and total cholesterol, determined upon admission to the hospital. The following scoring system was used for the calculation of the CONUT score: serum albumin ≥ 3.5 g/dL = 0 points, 3.0–3.4 g/dL = 2 points, 2.5–2.9 g/dL = 4 points, and <2.5 g/dL = 6 points; total cholesterol ≥ 180 mg/dL = 0 points, 140–179 mg/dL = 1 point, 100–139 mg/dL = 2 points, and <100 mg/dL = 3 points; total lymphocyte count ≥ 1600/mL = 0 points, 1200–1599/mL = 1 point, 800–1199/mL = 2 points, and <800/mL = 3 points [20,21]. A CONUT score < 3 indicates normal nutritional status or mild malnutrition, while a CONUT score ≥ 3 indicates moderate or severe risk of malnutrition.

**Figure 1:**
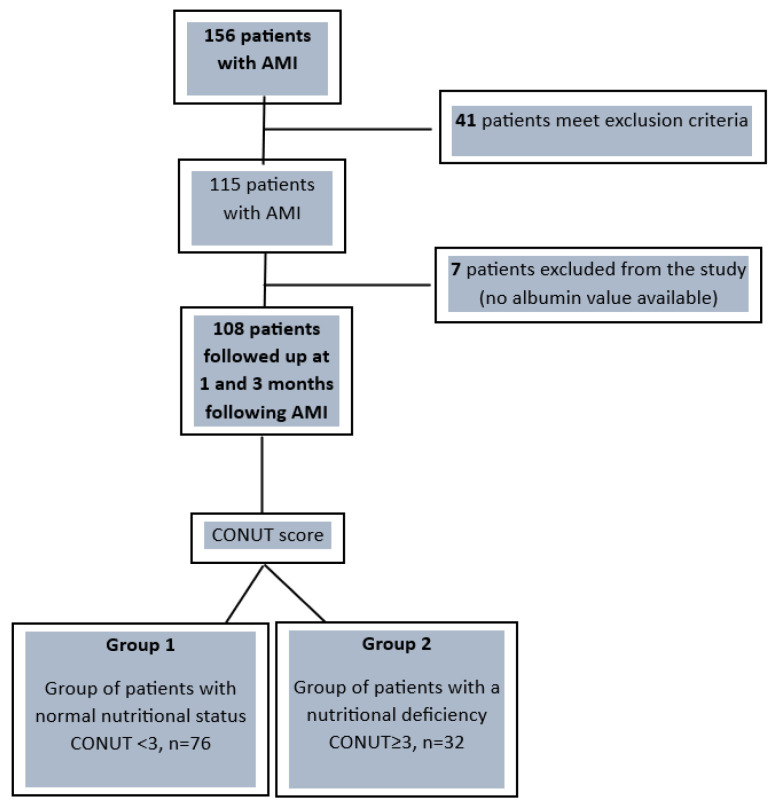
Overall study design flowchart. AMI—acute myocardial infarction; CONUT—Controlling Nutritional Status.

Based on the CONUT score, the 108 patients included in the final analysis were divided as follows: Group 1 with normal or mild nutritional deficit (CONUT < 3 points, *n* = 76), and Group 2 malnourished, with moderate to severe nutritional deficiency (CONUT ≥ 3, *n* = 32). Figure 1 illustrates the overall study design flowchart.

The MACE rate was determined for all patients at 1 and 3 months following AMI. The number of patients who presented MACE was obtained from the total number of patients and reported as total MACE, as well as the number of events, taking into account the fact that some patients presented more than one adverse event during the follow-up period. The following adverse events were included in the MACE category for the current study: cardiovascular death, non-fatal MI, resuscitated cardiac arrest, revascularization, ventricular arrhythmias, atrioventricular blocks requiring pacing, and ischemic stroke.

### Statistical Analysis

Data were analyzed using Graph Pad InStat version 3.10. (GraphPad Software, Inc., San Diego, CA, USA). All continuous variables are presented as mean ± standard deviation, while categorical variables are expressed as numbers and percentages. We used the unpaired Student’s *t*-test for normally distributed continuous variables to compare groups of individuals with normal nutritional status and those with nutritional deficiency. The Fischer’s exact test was used to compare categorical variables, and logistic regression analysis was performed to investigate the association between nutritional status as a function of CONUT score, inflammatory status, and cardiovascular events. The threshold for statistical significance was set at *p* < 0.05.

## 3. Results

A total of 108 patients were included in the current analysis, out of which Group 1 (*n* = 76) with a CONUT score < 3 had a normal nutritional status, and Group 2 (*n* = 32), with a CONUT ≥ 3 score, had a moderate to severe nutritional deficiency. There was no statistically significant difference between the two groups in terms of patients mean age (61.30 ± 13.37 years versus 62.38 ± 12.30 years; *p* = 0.68). Furthermore, there was no significant difference between the two groups in terms of weight, height, ideal weight, body mass index, nor medical history (all *p* > 0.05). The demographic and the medical history data are presented in Table 1.

**Figure 2:**
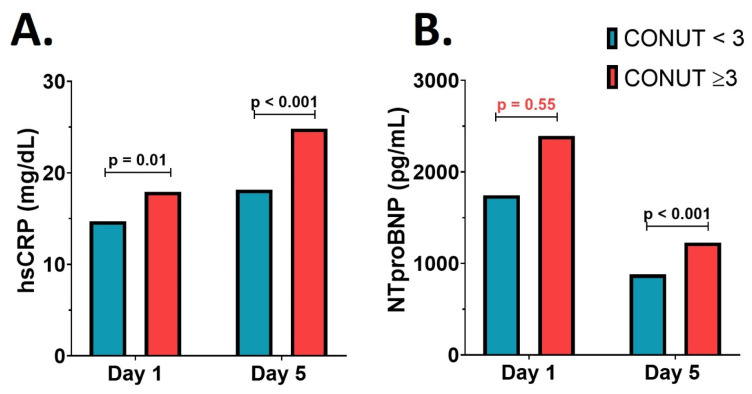
Serum inflammatory and left ventricular disfunction parameters. (**A**) The high-sensitivity *C*-reactive protein (hsCRP) value. (**B**) The *N*-terminal pro–B-type natriuretic peptide (NTproBNP) value.

The serum hsCRP level on the first day after de AMI was elevated in both groups. It was significantly higher in patients with a CONUT score ≥ 3 (17.94 ± 3.28 mg/dL versus 14.71 ± 3.35 mg/dL; *p* = 0.01); this difference remained significant on the fifth day after the AMI (24.82 ± 4.57 mg/dL versus 18.17 ± 3.38 mg/dL; *p* < 0.001)—Figure 2A. Although there were no significant differences between the groups on day 1 in terms of NTproBNP value as a marker of left ventricular dysfunction (2393.0 ± 956.0 pg/mL versus 1745.0 ± 570.8 pg/mL; *p* = 0.55), 5 days after the acute event, the NTproBNP value was significantly higher in patients with altered nutritional status (1227.0 ± 334.6 pg/mL versus 880.8 ± 139.0 pg/mL; *p* < 0.001)—Figure 2B. The LVEF was significantly lower in the malnourished group at baseline compared to the group with normal nutritional status, (48.53 ± 0.49% versus 42.37 ± 0.62%; *p* < 0.001).

Biochemical profile and blood cell count in patients with CONUT < 3 versus CONUT ≥ 3 at 1 month and 3 months follow-up are presented in Table 2. Paradoxically, the patients with a deficient nutritional status presented, mainly at 3 months follow-up, a better lipid profile, evidenced by a lower LDL-cholesterol and a lower triglyceride value, respectively, and a higher HDL-cholesterol compared to the patients with CONUT < 3 (all *p* < 0.05). Also, patients with CONUT ≥3 showed higher values of fasting blood glucose during the follow-up period.

We assessed the relationship between nutritional status and the occurrence of major cardiovascular events at both 1 month and 3 months following the AMI—Table 3. Patients with altered nutritional status (CONUT ≥ 3) experienced significantly higher rates of major cardiovascular events 1 month and 3 months following the acute event compared to patients with CONUT < 3 (both *p* < 0.0001). There were significant differences between the two groups in the occurrence of non-fatal MI (*p* < 0.001), resuscitated cardiac arrest (*p* = 0.02), revascularization (*p* = 0.02), ventricular arrhythmia (*p* = 0.007) and atrioventricular blocks (*p* < 0.001) at 1 month after the acute event, where more cases occurred in the malnourished group. The values are presented as absolute numbers and percentages. The values from the 3-month follow-up also include the values presented at the 1-month follow-up.

## 4. Discussion

Patients with cardiovascular disease are more likely to suffer from malnutrition. At the same time, malnutrition is linked to a longer stay in a medical unit, more hospitalizations and readmissions, a higher risk of treatment-related complications, and even an increased risk of death. It is thus a public health concern because it raises the cost of patient care [22,23,24]. Malnutrition status in AMI patients influences the number of complications and treatment outcomes [25,26]. The precise prevalence of malnutrition, however, is difficult to determine due to a lack of standardized diagnostic methods.

The present study aimed primarily to identify the impact of nutritional status on the occurrence of cardiovascular events at 1 month and 3 months after AMI. Malnourished patients, identified by a high CONUT score, showed higher levels of inflammation and more severe left ventricular dysfunction, as hsCRP and NTproBNP values at baseline and day 5 were higher in malnourished patients compared to those with normal nutritional status. According to a recent study by Sze S. et al. [27], who analyzed the occurrence and prognostic value of malnutrition in patients with heart failure (HF), malnutrition occurred more frequently in patients with elevated NTproBNP. This was also confirmed by our results; in addition, Sze S. et al. [27] showed that worse nutritional status seems to be associated with worse outcomes, independent of LVEF. Another aspect that can be observed in our study is that malnourished patients showed increased markers of blood vulnerability given by low lymphocyte values both at 1 month and 3 months after infarction.

Other studies suggested that in patients undergoing PCI, low serum albumin level, independent of the traditional risk factors, was associated with the occurrence of MACE [28,29]. In addition, these markers have been shown to have different associations with poor prognosis and mortality in similar disease groups.

In a retrospective study of 268 consecutive AMI patients who underwent percutaneous coronary intervention (PCI), also GNRI, as a marker of nutritional status, was identified as a potential predictor of mortality in AMI patients beyond one month after PCI, regardless of GRACE risk score [30]. Deng X. et al. published a study in 2020 that investigated the relationship between the CONUT score and the MACE rate in post-AMI patients who underwent percutaneous coronary intervention. The patients were divided into three groups based on their CONUT scores, and 751 patients were followed for about two years. The MACE rate in the severe nutritional deficiency group was 45.5%, compared to 6.1% in patients with a CONUT score of 0–1, indicating that patients with a CONUT score ≥ 5 had the significantly higher rates of MACE [31].

A systematic review of nine observational studies involving more than 80,257 patients with coronary artery disease found that malnutrition as defined by the CONUT score was associated with a significantly higher risk of all-cause mortality. Furthermore, each point increase in the CONUT score was associated with a 20% and 23% higher risk of mortality and MACE, respectively. All of these findings highlight the importance of clinicians detecting malnutrition in high-risk populations as early as possible. This improves risk stratification and helps guide future secondary preventive interventions [32].

In a study published in 2018 by Rus V. et al. [33], nutritional deficiency was linked to an increased rate of complications during hospitalization in patients with AMI. Patients with a CONUT score above three points were more likely to experience acute heart failure, hemodynamic instability, inotropic therapy, and a longer stay in the cardiac intensive care unit than those with a CONUT score < 3 points (normal to mildly deficient nutritional status) [33]. Screening for malnutrition in AMI patients may lead to fewer in-hospital complications, shorter lengths of stay, and lower healthcare costs [33].

The relationship between nutritional status, especially malnutrition, and the prognosis of patients with cardiovascular disease has sparked increased research interest [34]. Therefore, more precise research with a longer follow-up time for the patient with AMI, but also treatment and screening of malnutrition in cardiovascular disease would be of a great importance.

Given the high prevalence of malnutrition and the repercussions for morbidity–mortality of patients with cardiovascular disease and for healthcare costs, nutritional screening measures such as the calculation of nutritional scores should be placed as a first step in integrated nutritional care for patients during hospitalization and beyond [7].

In our study, cardiovascular events occurred more frequent in those with impaired nutritional status (total MACE—46.9% versus 9.2% at 1 month follow-up and 68.8% versus 10.5% at 3 months follow-up), who more often presented events such as non-fatal MI, stroke, revascularization, ventricular arrhythmia and cardiovascular death. While non-fatal MI occurred more frequently at 1 month than at 3 months, (17.6% versus 13%), stroke, revascularization, ventricular arrhythmia and atrioventricular block are more frequent at 3 months post AMI.

The current study’s findings must also be considered in light of some limitations. Firstly, the study population consists of a small number of AMI patients recruited from a single Cardiology site, and the follow-up period was a short one. The extension to a longer period of follow-up would increase the value of the analysis. Secondly, we used only one of the nutrition status screening tools, the CONUT score, and we did not compare it with other tools, such as PNI, GNRI. The advantage of the COUNT score is that it includes the most serum nutritional indicators. Compared to the PNI, the COUNT score considers the impact of total cholesterol on nutritional status. While the GNRI is a modification of the nutritional risk index tailored to geriatric patients, in our case, the studied population does not include only geriatric individuals, so we chose the CONUT score.

Thirdly, inflammatory status was assessed using only hsCRP, a standard biomarker associated with systemic inflammation, without using other biomarkers such as interleukins. While interleukins and other biomarkers can provide additional insights into inflammatory processes, hsCRP remains a widely accepted marker for assessing overall inflammatory status. The choice of biomarkers may vary depending on the specific research or clinical context, but hsCRP alone can still offer valuable information about inflammation levels in many situations [35].

## 5. Conclusions

Following an AMI, patients with poor nutritional status experienced a higher inflammatory status frailty and cardiovascular events more often than those with normal nutritional status. Use of the CONUT nutritional score in conjunction with inflammatory biomarkers demonstrated a strong association with poor prognosis in malnourished patients with cardiovascular disease. The link between the nutritional status and the appearance of MACE in post-AMI patients might be the increased inflammatory status, which is at least partly maintained by the nutritional deficit. However, additional studies are needed to reveal mechanism which drives the triad of nutritional status—inflammation—cardiovascular events. These results suggest that a personalized nutritional treatment after an accurate assessment of the nutritional status might lead to a better outcome in patients following AMI. It is crucial for healthcare professionals to assess and address the nutritional needs of patients with AMI to support their recovery and reduce the risk of complications. This may involve working with dietitians or nutritionists to develop personalized dietary plans that meet the patient’s nutritional requirements while also supporting heart health. Additionally, addressing any underlying psychological or functional issues that may affect dietary intake is essential for promoting overall well-being during recovery from a heart attack.

## Figures and Tables

**Table 1:** Demographic data and medical history in patients with CONUT < 3 *versus* CONUT ≥ 3.

	CONUT < 3 (*n* = 76)	CONUT ≥ 3 (*n* = 32)	*p* Value	R.R.
Demographic data				
Age (years)	61.30 ± 13.37	62.38 ± 12.30	0.68	N.A.
Weight (kg)	72.53 ± 5.20	70.81 ± 6.15	0.17	N.A.
Height (m)	1.73 ± 0.13	1.71± 0.15	0.51	N.A.
BMI (kg/m^2^)	24.25 ± 2.53	24.23 ± 2.40	0.95	N.A.
Medical history				
CHD	24 (31.58%)	11 (34.37%)	0.95	0.92
Hypertension	52 (68.42%)	28 (87.50%)	0.06	0.78
Ischemic stroke	9 (11.84%)	3 (9.38%)	0.97	1.26
Diabetes mellitus	12 (15.79%)	7 (21.87%)	0.63	0.72

The results are expressed as mean ± SD and absolute value (percentage). *p*-values refer to between-group comparisons based on the *t*-test or chi-squared test. R.R.—relative risk; BMI—body mass index; CHD—coronary heart disease.

**Table 2:** Biochemical profile and blood cell count in patients with CONUT < 3 versus CONUT ≥ 3 at 1 month and 3 months follow-up.

	1 Month Follow-Up			3 Months Follow-Up		
	CONUT < 3 (*n* = 76)	CONUT ≥ 3 (*n* = 32)	*p* Value	CONUT < 3 (*n* = 76)	CONUT ≥ 3 (*n* = 32)	*p* Value
Biochemical profile						
LDL cholesterol (mg/dL)	87.22 ± 37.00	65.57 ± 12.95	**<0.001**	65.94 ± 22.936	40.67 ± 15.670	**<0.001**
HDL cholesterol (mg/dL)	48.33 ± 14.35	40.79 ± 13.56	0.06	55.25 ± 13.894	67.15 ± 26.733	**<0.001**
Triglycerides (mg/dL)	151.54 ± 92.98	126.31 ± 82.95	0.17	131.53 ± 121.02	80.85 ± 60.97	**<0.001**
Fasting blood glucose (mg/dL)	124.29 ± 39.22	154.96 ± 89.32	**0.01**	113.30 ± 21.01	124.67 ± 39.21	0.053
Urea (mg/dL)	42.12 ± 32.12	47.24 ± 37.42	0.50	37.58 ± 26.80	57.56 ± 37.35	**<0.001**
Creatinine (mg/dL)	1.06 ± 0.40	1.10 ± 0.59	0.76	1.05 ± 0.47	1.19 ± 0.39	0.14
AST (mg/dL)	47.80 ± 63.00	43.83 ± 60.38	0.76	21.67 ± 11.33	18.04 ± 5.25	**0.02**
ALT (mg/dL)	45.14 ± 61.16	46.03 ± 62.80	0.94	44.84 ± 29.39	30.41 ± 12.65	**<0.001**
GGT (mg/dL)	56.27 ± 31.65	64.00 ± 33.02	0.82	54.69 ± 48.19	39.67 ± 7.13	**0.01**
Uric acid (mg/dL)	5.69 ± 1.71	5.40 ± 2.17	0.53	5.27 ± 1.98	5.67 ± 2.13	0.40
CK (U/L)	371.31 ± 623.44	197.19 ± 252.45	0.08	157.37 ± 91.49	171.52 ± 99.92	0.50
Cl^−^ (mmol/L)	103.26 ± 2.62	103.27 ± 1.34	0.98	101.14 ± 3.56	102.04 ± 3.65	0.27
K^+^ (mmol/L)	4.35 ± 0.43	4.34 ± 0.41	0.90	4.53 ± 0.50	4.59 ± 0.50	0.54
Na^+^ (mmol/L)	139.84 ± 2.81	139.29 ± 3.27	0.42	138.43 ± 2.42	137.19 ± 2.11	**0.01**
Blood cell count						
WBC (×10^3^/mm^3^)	10.25 ± 3.37	10.49 ± 4.80	0.80	8.27 ± 2.12	7.52 ± 0.70	**<0.001**
RBC (×10^6^/mm^3^)	4.61 ± 0.65	4.46 ± 0.65	0.29	4.91 ± 0.39	4.48 ± 0.97	**<0.001**
Hb (g/dL)	14.34 ± 1.63	13.97 ± 2.35	0.42	14.90 ± 1.40	13.56 ± 2.70	**<0.001**
Hct (%)	43.56 ± 8.10	41.33 ± 6.54	0.14	43.57 ± 4.22	40.59 ± 7.89	**0.01**
PLT (×10^3^/mm^3^)	246.77 ± 65.05	271.34 ± 92.90	0.18	237.47 ± 55.92	245.78 ± 68.78	0.53

The results are expressed as mean ± SD. *p*-values refer to between-group comparisons based on the *t*-test. *p*-values less than 0.05 are highlighted in bold. LDL—low-density lipoprotein; HDL—high-density lipoprotein; AST—aspartate aminotransferase; ALT—alanine transaminase; GGT—gamma-glutamyl transferase; CK—creatine kinase; WBC—white blood cells; RBC—red blood cells; Hb—hemoglobin; Hct—hematocrit; PLT—platelets.

**Table 3:** The relationship between nutritional status expressed by the CONUT score and major cardiovascular adverse events at 1 month follow-up and 3 months follow-up, respectively.

Major CV Adverse Events	1 Month Follow-Up			3 Months Follow-Up		
	CONUT < 3 (*n* = 76)	CONUT ≥ 3 (*n* = 32)	*p* Value	CONUT < 3 (*n* = 76)	CONUT ≥ 3 (*n* = 32)	*p* Value
Number of patients presenting MACE						
Total MACE (%)	7 (9.2%)	15 (46.9%)	**<0.0001**	8 (10.5%)	22 (68.8%)	**<0.0001**
Number of events	**(*n* = 7)**	**(*n* = 31)**		**(*n* = 17)**	**(*n* = 38)**	
CV death (%)	0	0	N.A.	1	3	**0.04**
Non-fatal MI (%)	7	12	**<0.001**	5	9	**<0.001**
Resuscitated cardiac arrest (%)	0	3	**0.02**	3	3	0.16
Revascularization (%)	0	3	**0.02**	0	4	**0.003**
Ventricular arrhythmias (%)	0	4	**0.007**	1	6	**<0.001**
AV blocks (%)	0	9	**<0.001**	4	9	**<0.001**
Ischemic troke (%)	0	0	N.A.	3	4	0.06

The results are expressed as absolute values and/or percentages. Total MACE—refers to the number of patients who presented adverse events. Each type of adverse event is reported as the number of events that occurred. *p*-values refer to between-group comparisons based on Fisher’s exact test. *p*-values less than 0.05 are highlighted in bold. CV—cardiovascular; MACE—major cardiovascular adverse events; MI—myocardial infarction; AV—atrio-ventricular.

## Data Availability

The raw data supporting the conclusions of this article will be made available by the authors on request.
